# Evaluation of the total distribution volume of ^18^F-FBPA in normal tissues of healthy volunteers by non-compartmental kinetic modeling

**DOI:** 10.1007/s12149-019-01427-9

**Published:** 2019-12-05

**Authors:** Victor Romanov, Kayako Isohashi, Galal Alobthani, Rouaa Beshr, Genki Horitsugi, Yasukazu Kanai, Sadahiro Naka, Tadashi Watabe, Eku Shimosegawa, Jun Hatazawa

**Affiliations:** 1 Department of Nuclear Medicine and Tracer Kinetics, Osaka University Graduate School of Medicine, Yamada Oka 2-2, Suita, Osaka 565-0871 Japan; 2 Immunology Frontier Research Center, Osaka University, Suita, Japan; 3 Department of Molecular Imaging in Medicine, Osaka University Graduate School of Medicine, Suita, Japan

**Keywords:** FBPA, BPA, Boron concentration, Total distribution volume

## Abstract

**Objective:**

Boron neutron capture therapy (BNCT) is a noninvasive radiation therapy method for cancer treatment. In BNCT, 4-borono-2-[^18^F]-fluoro-L-phenylalanine (^18^F-FBPA) PET has been employed to estimate ^10^B accumulation in target tumors and normal tissues if ^10^B borono-L-phenylalanine (^10^B-BPA) is used as a boron carrier. The purpose of the current study was to evaluate the total distribution volume (Vt) of ^18^F-FBPA in normal organs of healthy volunteers by kinetic analysis and to estimate boron concentration in normal organs for the therapeutic dose of ^10^B-BPA using obtained Vt values.

**Methods:**

Six healthy volunteers were injected with ^18^F-FBPA (3–5 MBq/kg), and 7 PET-CT scans were performed subsequently. ^18^F-FBPA radioactivity in whole blood and plasma was measured before, and eight times after the injection. PET images were analyzed by PMOD software. Twelve volumetric regions of interest including the brain, heart, right lung, spleen, liver, parotid salivary glands, esophagus, stomach, pancreas, intestines, and bone marrow were drawn manually for each subject and analyzed with the Logan plot and two Ichise multilinear analyses (MA1 and MA2). The better model was defined by several goodness-of-fit parameters and residual distribution. After Vt values had been derived, boron concentration was estimated in ppm for the ^10^B-BPA-fructose (^10^B-BPA-fr) dose 30 g 1 and 2 h post-injection using Vt and interpolated plasma activity data.

**Results:**

The Ichise MA2 model showed the best fit among all models. Akaike Information Criterion (AIC) was the lowest for the Ichise’s MA2 in all regions (mean AIC value − 14.0) comparing to the other models (Logan plot mean AIC 31.4; Ichise MA1 model mean AIC − 4.2). Mean Vt values of the Ichise MA2 model ranged from 0.94 ± 0.14 ml/ml in the pancreas to 0.16 ± 0.02 ml/ml in the right lung. Estimated boron concentration for ^10^B-BPA-fr had the highest value in the pancreas (14.0 ± 1.9 ppm 1 h after, and 5.7 ± 1.7 ppm 2 h after the ^18^F-FBPA administration) and the lowest value in the right lung (2.4 ± 0.3 ppm 1 h, and 1.0 ± 0.3 ppm 2 h post-injection).

**Conclusion:**

The ^10^B concentration in normal tissues was best estimated using Vt values of ^18^F-FBPA with the Ichise multilinear analysis 2 (MA2).

**Trail registry:**

The UMIN clinical trial number: UMIN000022850.

**Electronic supplementary material:**

The online version of this article (10.1007/s12149-019-01427-9) contains supplementary material, which is available to authorized users.

## Introduction

Boron neutron capture therapy (BNCT) is a rapidly growing area of medicine, at the junction of radionuclide diagnostics, nuclear physics, radiation therapy and, pharmacology. However, uptake of the boron-carrying molecules in target cells is heterogeneous [1–3], depends on cellularity [4], cell cycle phase [5, 6], and many other factors. For effective treatment, the ratio of ^10^B concentration in the tumor and its concentration in normal tissues (T/N ratio) should be more than 3:1, and the ^10^B concentration in the target should be at least 15 μg/g [7, 8]. Therefore, to avoid adverse effects, it is necessary to know the concentration of the ^10^B not only in tumor cells but also in normal tissues.

One of ^10^B carriers in BNCT is ^10^B 4-borono-L-phenylalanine (^10^B-BPA). PET with 4-borono-2-[^18^F]-fluoro-L-phenylalanine (^18^F-FBPA) can be used for estimation of ^10^B concentration in vivo [9] because ^18^F-FBPA molecule has similar pharmacological behavior in normal tissues to that of ^10^B-BPA [10, 11].

There are several methods of evaluation of ^10^B concentration in tumors and normal tissues through ^18^F-FBPA PET. It could be estimated through the method proposed by Shimosegawa [12] in ppm. This method demonstrated a good correlation between predicted and actual boron concentrations in brain tissue. However, ^18^F-FBPA injection protocol used in PET studies differs from the therapeutic protocol of ^10^B-BPA administration, which could result in a discrepancy between the estimated and actual values of ^10^B concentration.

The purpose of the current study was to evaluate ^18^F-FBPA total distribution volume (Vt) in normal organs and tissues of healthy volunteers by non-compartmental, tracer reversible-binding graphical analysis of ^18^F-FBPA PET images and to estimate boron concentration in normal tissues in human with obtained Vt values.

There are several studies performed in the laboratory of Nuclear Medicine Department of Osaka University concerning ^18^F-FBPA properties and application in BNCT at present. Hanaoka et al. found a significant correlation between ^10^B-BPA-fructose and ^18^F-FBPA concentrations in an animal model. Shimosegawa et al. [12] could estimate boron concentration in ppm in normal tissues of healthy volunteers for ^18^F-FBPA and therapeutic doses of ^10^B-BPA-fructose complex (^10^B-BPA-fr). Watabe et al. [13] proposed an approach to estimate boron concentration in absolute units form SUV of ^18^F-FBPA-PET data using a rat model. Isohashi et al. [14] compared image-derived radioactivity and blood-sample radioactivity to be able to estimate boron concentration in the tumor before BNCT.

## Materials and methods

### ^18^F-FBPA synthesis

^18^F-FBPA was prepared following the method of direct radio fluorination of ^10^B-BPA, proposed by Ishiwata et al. [15] with a number of modifications, using the F-1 synthesizer (Sumitomo Heavy Industries, Tokyo, Japan).

First, elemental ^18^F_2_ gas was produced in ^20^Ne(d,α)^18^F reaction and converted to ^18^F-acetylhypofluorite in reaction with sodium acetate. ^18^F-acetylhypofluorite was bubbled at a flow rate of 600 ml/min into 5 ml of trifluoroacetic acid containing 30 mg of 4-borono-L-phenylalanine, at room temperature. Then, trifluoroacetic acid was removed by passing N_2_ under reduced pressure at a flow rate of 200 ml/min. Next, the residue was dissolved in 3 ml of 0.1% acetic acid, and the solution was passed through a high-performance liquid chromatography column YMC-Pack ODS-A (YMC, Kyoto, Japan) 20 × 150 mm, with the flow rate of 10 ml/min and 0.1% acetic acid as a mobile phase. Ultraviolet detector for wavelength 280 nm and radioactivity detector were applied for monitoring of the elution profile. After the ^18^F-FBPA fraction was collected (retention time from 19 to 21 min), resultant ^18^F-FBPA radiochemical purity was > 98%. The specific radioactivity was 44.1 ± 4.9 GBq/mmol.

### The study protocol

The study involved 6 volunteers: 4 males and 2 females between ages 21 and 56 (average age 33 ± 16 years), and with the weight range of 48–66 kg (mean weight 61 ± 7 kg). None of the subjects had a prior history of any major diseases, drug or alcohol abuse, and cigarette smoking. All procedures of the study were explained in detail, and signed informed consent was collected from all subjects. The study was performed under the regulations of the institutional ethics committee for clinical research of Osaka University (No. 12113).

Subjects were prepared for the study by 4 h of fasting. After that, ^18^F-FBPA (3–5 MBq/kg) was injected as a bolus into the left cubital vein. Subsequently, seven repeated whole-body PET scans from the top of the head to the mid-thigh were performed. The decay of radioactivity during the PET scan was corrected to the start time of the first scan. The data consisted of seven scans with 455 s acquisition time and the interval between scans of 45–46 s. ^18^F-FBPA radioactivity in whole blood and plasma was measured at the background, 30 s, 1 min, 3 min, 5 min, 10 min, 20 min, 30 min, and 50 min using the PET camera and the well counter (Shimadzu Co, Kyoto, Japan), corrected mutually. The concentration of ^18^F-FBPA metabolites in blood was also determined at the 20th and 50th minutes with mean values of 2.324% and 3.966% respectively, showing high stability. A whole-body CT scan (140 kV, 120–240 mAs) was performed after PET, for image fusion. Obtained images were reconstructed by Dynamic Row-Action Maximum Likelihood Algorithm (DRAMA) with an image matrix of 128 × 128, and a voxel size of 4.0 × 4.0 × 3.25 mm^3^. The axial field of view was 26 cm. All PET-CT studies were performed on Eminence SOPHIA SET-3000BCT/X scanner (Shimadzu Corporation) in the period from July 2013 to October 2013.

### Image analysis

PET images were analyzed with PMOD biomedical image quantification software, build 3.601. Interpolation of the FBPA time-activity curves in plasma was carried out by the JMP Pro 13.0.0 software. Simple statistical calculations (mean, standard deviation values, and graphs) were carried out by the Microsoft Excel 2013 program.

Measurement of ^18^F-FBPA accumulation was implemented through applying volumetric regions of interest (VOI) on 12 areas: the brain, heart, right lung, spleen, liver, parotid salivary glands on both sides, esophagus, stomach, pancreas, intestines, bone marrow. VOI setting was performed manually on each cross-sectional ^18^F-FBPA PET image with CT images used as the reference. Tracer accumulation in bone marrow was evaluated by placing VOI on lumbar spine vertebrae bodies L1–L5. Tracer activity in both parotid glands was analyzed as one area. Vt quantification was carried out by two non-compartmental methods: the Logan plot graphical analysis using a single linear regression model, and two Ichise multilinear analysis (MA1 and MA2 models).

Following equations were employed for Vt calculation:

*Logan plot*

$$\frac{{\int_{0}^{T} C \left( t \right){\text{d}}t}}{C(T)} = {\text{Vt}}\int_{0}^{T} {\frac{{{\text{Cp}}\left( t \right){\text{d}}t}}{C(T)}} + b$$

*Ichise analysis*

MA1 $$C\left( T \right) = - \frac{\text{Vt}}{b}\int_{0}^{T} {\text{Cp}} \left( t \right){\text{d}}t + \frac{1}{b}\int_{0}^{T} C \left( t \right){\text{d}}t$$

MA2 $$C\left( T \right) = \gamma_{1} \int_{0}^{T} {\int_{0}^{S} {\text{Cp}} } \left( t \right){\text{d}}t{\text{d}}s + \gamma_{2} \int_{0}^{T} {\int_{0}^{S} C } \left( t \right){\text{d}}t{\text{d}}s + \gamma_{3} \int_{0}^{T} C \left( t \right){\text{d}}t + \gamma_{4} \int_{0}^{T} {\text{Cp}} \left( t \right){\text{d}}t,$$

where *C*(*t*) is the radioactivity concentration (kBq/ml) in VOI at time *t* (measured by PET); Cp(*t*) is the radioactivity concentration (kBq/ml) in plasma at time *t* (measured by the blood sampling corrected for metabolites); the slope (Vt) is the total distribution volume (ml/ml); *b* is the intercept; *γ*_1–4_ are expressions including two-tissue model rate constants: *γ*_1_ = *k*_2_/*k*_4_*V*; *γ*_2_ = −*k*_2_/*k*_4_, *γ*_3_ = −(*k*_2_ + *k*_3_ + *k*_4_), *γ*_4_ = *k*_1_ [16, 17].

*k*_1_ and *k*_2_ represent the rate constants for the first tissue compartment, where *k*_1_ characterizes the transport of the tracer from the blood to the tissue compartment, and *k*_2_ characterizes the transport of the tracer to the opposite direction, from the first tissue compartment to the blood. And *k*_3_, *k*_4_ are the rate constants in 2 tissue compartment model and represent inflow of the tracer in the second tissue compartment and its outflow from the second to the first tissue compartment.

An equilibration time t* (the time after which the plots of the equations become linear) with the maximal allowed error of the regression 1% was set automatically for the Logan plot and for the Ichise MA1 and was fixed to 20 min for MA2 in all regions, since no equilibration is required for this model. The more suitable model had been selected on the ground of the Akaike Information Criterion (AIC) and reduced Chi-square (Chi-square) as goodness-of-fit parameters. Also, the coefficient of determination (*R*^2^) was assessed, and residual distribution was analyzed and compared by the sum of squared residuals and the standard deviation of the residuals (Sy.x). ^18^F-FBPA activity in plasma was used as the input function. Tracer concentration in kBq/ml was used for input function and tissue data import, under the assumption that the volume of 1 g of a tissue equals 1 ml. Subsequently, Vt values were calculated for each organ using the better model.

To estimate boron concentration for the conditions of the therapeutic BNCT protocol with ^10^B-BPA-fructose injection dose 30 g (500 mg/kg; BW 60 kg) 1 and 2 h after ^18^F-FBPA injection, first, the data set of plasma tracer activity in kBq/ml was derived for each subject using plasma activity curves interpolated following tracer clearance data points with a bi-exponential model (Online Resource 1). Next, ^18^F-FBPA tissue activity in kBq/ml was calculated in all VOI of each subject by the formula:

1 $$T = P*V,$$

where *T* is the ^18^F-FBPA concentration in an organ (kBq/ml); *P* is the ^18^F-FBPA concentration in plasma in a given time (kBq/ml), and *V* is the total distribution volume for that organ.

After that, ^18^F-FBPA tissue concentrations were represented as molar concentrations:

2 $$M = \frac{T}{S},$$

where *M* is the ^18^F-FBPA tissue molar concentration in mol/ml; *T* is the ^18^F-FBPA activity tissue concentration in kBq/ml; *S* is the ^18^F-FBPA mean specific radioactivity (4.41 × 10^10^ kBq/mol).

Then, boron concentrations in ppm were calculated as follows, proceeding from the fact that one ^18^F-FBPA molecule contains one ^10^B atom, and averaged for each VOI:

4 $$B = {\text{MW}}_{\text{B}} *M* \, 10^{6} ,$$

where *B* is the boron concentration in ppm; MW_B_ is the boron molecular weight (10 g/mol); *M* is ^18^F-FBPA tissue molar concentration in mol/ml.

Finally, boron concentration was estimated for the ^10^B-BPA-fr therapeutic dose 30 g, with the formula devised by Shimosegawa et al. [12].

5 $$H = B*\frac{{30\,{\text{g}}}}{I}*\frac{\mathrm{MWfbpa}}{\mathrm{MWbpa}},$$

where *H* is ^10^B fraction for ^10^B-BPA-fr dose 30 g in ppm; *B* is the ^10^B fraction of ^18^F-FBPA (ppm); *I* is the mean ^18^F-FBPA injected dose in grams (0.00102 g); MWfbpa is the ^18^F-FBPA molecular weight (226.9 g/mol); MWbpa is the ^10^B-BPA-fr molecular weight (389.3 g/mol).

## Results

Tables 1, 2, and 3 display goodness of fit parameters employed to evaluate and compare the models. Multilinear models demonstrated relatively better goodness of fit than the single linear model, and the MA2 model appeared to be more accurate than MA1. AIC (the smaller the value, the better the fitting) showed more accurate fitting of the model curve for the MA2 model in all regions (Table 1). Chi-square (the value closest to 1 imply the better fitting) results were comparable for both MA1 and MA2 Ichise models, however, underestimation of the error was observed in most regions of MA2 and half of all areas of the MA1 model (the other half showed overestimation of the error) (Table 2). Sy.x (the value closest to 0 implies the better fitting) had more appropriate results for the MA2 model (9 VOIs out of 11), indicating better model compliance for this model (Table 3). In the case of the MA2 model, *R*^2^ was > 0.9 for all of the VOI except for the brain (mean *R*^2^ 0.8), showing a good correlation between the explanatory and dependent variables. For the Logan plot, all parameters demonstrated significantly more poor results than for the multilinear models (Tables 1, 2, 3).

**Table 1:** Mean and one standard deviation of Akaike information criterion (AIC) for the Ichise MA1 and MA2 models

AIC			
VOI	Ichise MA2	Ichise MA1	Logan plot
Pancreas	− 9.18 ± 6.50	− 8.65 ± 5.93	34.00 ± 6.08
Liver	− 17.66 ± 10.96	− 1.76 ± 7.55	26.29 ± 6.62
Salivary glands	− 6.01 ± 3.09	− 1.90 ± 4.16	32.22 ± 6.40
Esophagus	− 11.97 ± 6.23	− 5.39 ± 1.02	31.79 ± 4.63
Stomach	− 17.11 ± 26.24	− 0.75 ± 7.81	30.08 ± 6.43
Heart	− 28.51 ± 13.96	− 5.26 ± 3.86	32.50 ± 5.59
Bone marrow	− 6.03 ± 5.48	0.73 ± 6.48	35.22 ± 2.55
Intestines	− 9.97 ± 11.50	0.17 ± 3.87	31.32 ± 4.04
Spleen	− 9.58 ± 7.39	− 4.22 ± 4.29	33.83 ± 3.19
Brain	− 24.27 ± 21.79	− 14.07 ± 5.75	29.76 ± 6.78
Lung	− 13.28 ± 11.03	− 4.57 ± 8.66	28.51 ± 7.59

**Table 2:** Mean and one standard deviation of Chi-square for the Ichise MA1, MA2 and the Logan plot models

VOI	Chi-square		
	Ichise MA2	Ichise MA1	Logan plot
Pancreas	0.41 ± 0.63	0.19 ± 0.26	264.55 ± 202.61
Liver	0.15 ± 0.21	3.51 ± 5.54	107.34 ± 165.67
Salivary glands	0.39 ± 0.33	1.20 ± 1.87	280.81 ± 92.96
Esophagus	0.17 ± 0.14	0.14 ± 0.04	168.25 ± 104.57
Stomach	0.68 ± 0.76	4.71 ± 6.20	133.33 ± 113.55
Heart	0.03 ± 0.04	0.21 ± 0.19	249.79 ± 274.13
Bone marrow	0.52 ± 0.49	3.72 ± 4.31	323.18 ± 267.74
Intestines	1.92 ± 4.06	1.78 ± 2.55	161.70 ± 163.90
Spleen	0.50 ± 0.87	0.56 ± 0.92	255.10 ± 269.65
Brain	0.11 ± 0.18	< 0.01 ± 0.01	143.00 ± 114.54
Lung	0.23 ± 0.27	3.12 ± 6.30	123.59 ± 111.02

**Table 3:** Standard deviation of the residuals (Sy.x) for the Ichise MA1, MA2 and the Logan plot models

VOI	Sy.x		
	Ichise MA2	Ichise MA1	Logan plot
Pancreas	0.18 ± 0.16	0.13 ± 0.12	14.69 ± 6.98
Liver	0.07 ± 0.06	0.34 ± 0.33	7.84 ± 6.77
Salivary glands	0.16 ± 0.04	0.22 ± 0.16	16.51 ± 2.85
Esophagus	0.09 ± 0.05	0.09 ± 0.03	12.21 ± 4.38
Stomach	0.17 ± 0.19	0.33 ± 0.28	10.16 ± 5.49
Heart	0.03 ± 0.03	0.10 ± 0.07	13.76 ± 7.78
Bone marrow	0.12 ± 0.05	0.30 ± 0.22	16.88 ± 6.18
Intestines	0.14 ± 0.19	0.21 ± 0.20	11.39 ± 5.65
Spleen	0.11 ± 0.12	0.11 ± 0.08	14.36 ± 7.00
Brain	0.02 ± 0.02	0.01 ± 0.01	10.38 ± 5.93
Lung	0.03 ± 0.02	0.06 ± 0.07	9.65 ± 5.52

Mean total volume of distribution values acquired by the Ichise model with MA2, MA1 equations and the Logan plot for each VOI are presented in Table 4.

**Table 4:** Averaged total volume of distribution values and one standard deviation for the Ichise MA1, MA2 and the Logan plot models

VOI	Mean Vt (ml/ml)		
	Ichise MA2	Ichise MA1	Logan plot
Pancreas	0.94 ± 0.14	0.92 ± 0.12	0.93 ± 0.14
Liver	0.81 ± 0.09	0.80 ± 0.08	0.80 ± 0.09
Salivary glands	0.74 ± 0.29	0.76 ± 0.09	0.76 ± 0.10
Esophagus	0.69 ± 0.12	0.70 ± 0.06	0.69 ± 0.06
Stomach	0.61 ± 0.24	0.63 ± 0.16	0.63 ± 0.17
Heart	0.59 ± 0.07	0.59 ± 0.06	0.59 ± 0.06
Bone marrow	0.55 ± 0.08	0.55 ± 0.07	0.55 ± 0.08
Intestines	0.55 ± 0.14	0.54 ± 0.11	0.54 ± 0.12
Spleen	0.53 ± 0.06	0.51 ± 0.04	0.51 ± 0.04
Brain	0.39 ± 0.05	0.37 ± 0.05	0.36 ± 0.05
Lung	0.16 ± 0.02	0.16 ± 0.02	0.15 ± 0.02

**Fig. 1:**
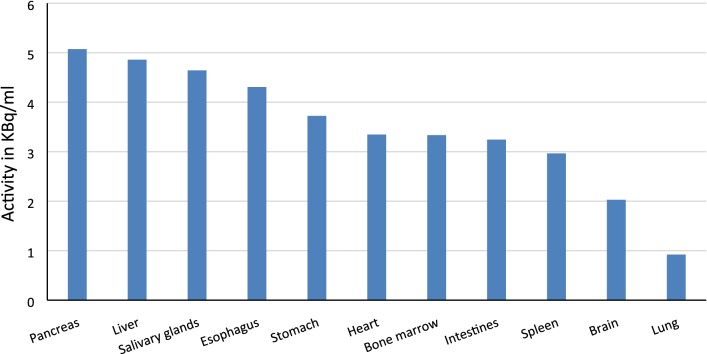
^18^F-FBPA activity in all selected VOIs in the washout phase (from the seventeenth minute), measured in kBq/ml and averaged for all subjects (*n* = 6). The highest value is detected in the pancreas, and the lowest in the lung. The mean activity is consistent with Vt values

Vt showed greater variability of the results between the subjects for the MA2 model, comparing to the MA1 model and the Logan plot, probably because of the constant t* 20 min for all VOIs (mean SD 0.8 for MA1 and the Logan plot vs. 0.12 for MA2). The highest average Vt value was observed in the pancreas, followed by liver, parotid glands, esophagus, stomach, heart, bone marrow, intestines, spleen, brain, and lung in descending order. In general, Vt values of ^18^F-FBPA in selected organs and tissues were consistent with its mean activity concentration in the washout phase. Data is represented in Table 4 and Fig. 1.

Estimated boron concentrations for the therapeutic ^10^B-BPA-fructose administration protocol at 1 and 2 h are presented in Table 5. It showed the maximal value in the pancreas (14.0 ± 1.9 ppm 1 h after, and 5.7 ± 1.7 ppm, 2 h after the ^18^F-FBPA administration) and the minimal value in the right lung (2.4 ± 0.3 ppm 1 h, and 1.0 ± 0.3 ppm 2 h post-injection).

**Table 5:** Boron concentration in selected VOIs, plasma, and whole blood estimated for the therapeutic ^10^B-BPA-fr BNCT protocol in ppm

VOI	Boron concentration (ppm)	
	1 h	2 h
Pancreas	14.0 ± 1.9	5.7 ± 1.7
Liver	12.1 ± 1.7	4.9 ± 1.4
Salivary glands	11.1 ± 1.5	4.5 ± 1.3
Esophagus	10.3 ± 1.4	4.2 ± 1.2
Stomach	9.1 ± 1.3	3.7 ± 1.1
Heart	8.8 ± 1.2	3.6 ± 1.0
Bone marrow	8.2 ± 1.1	3.4 ± 1.0
Intestines	8.2 ± 1.1	3.4 ± 1.0
Spleen	7.9 ± 1.1	3.2 ± 0.9
Brain	5.8 ± 0.8	2.4 ± 0.7
Lung	2.4 ± 0.3	1.0 ± 0.3
Plasma	14.9 ± 2.2	6.1 ± 1.9
Whole blood	11.4 ± 2.1	4.9 ± 1.0

The concentration is calculated at 1 and 2 h using Vt values and interpolated plasma ^18^F-FBPA activity values

## Discussion

In the current work, we estimated Vt of ^18^F-FBPA in healthy volunteers using single linear (Logan graphical analysis) and multilinear (Ichise MA1; MA2) models for reversibly binding tracers. This approach has several advantages, such as good reproducibility of the results in an individual patient or between the patients. Also, boron concentration could be estimated at any time point, after the equilibrium state between the influx of the tracer into the tissue compartment and its efflux to the plasma is reached.

The volume of distribution represents the ratio of the tracer concentrations in the target tissue (*Ct*) and plasma (Cp) at equilibrium or steady-state: Vt = *Ct*/Cp. Thereby knowing tracer concentration in plasma at equilibrium and Vt value for the certain tissue, *Ct* value for that tissue can be calculated. It is also independent of the tracer administration protocol in case if the constant ratio of tracer concentrations in blood and tissues is achieved [18], and could be applied to any reversibly binding tracer. ^18^F-FBPA uptake in normal tissues is reversible, it is not trapped inside the cells nor incorporated into proteins, and rapidly washed out from the tissues [10], and thus dynamic equilibrium between plasma and tissue ^18^F-FBPA concentrations is reached with time. Vt values obtained for ^18^F-FBPA could be applied for ^10^B-BPA, so finally tissue ^10^B-BPA concentration could be derived from its plasma concentration.

Initially, only the single linear analysis had been selected for its relative computational simplicity. However, the Logan plot was found to have low compatibility of the model and actual curves. It tended to show better fit with t* shifted manually to the later time points, partially due to the reduction of available data and lower variability. If t* value was changed to 33 min, where the model fit was acceptable, the remaining 3 data points were not sufficient to evaluate residual distribution adequately. Besides, stabilization of *γ*-intercept, that should become constant after the time t*, couldn’t be achieved due to limited data points. For that reason, multilinear models Ichise MA1 and MA2 had been chosen additionally.

Ichise multilinear analysis being a derivate of the Logan plot analysis provides reduced bias for both one-tissue and two-tissue compartment models, especially in case of region-of-interest based parameter estimation. MA1 is independent of t* only for a 1-tissue model, but as the Logan plot, it requires it for a 2-tissue model. MA2 is especially interesting because it does not require definite t* and could be applied for 1-tissue and 2-tissue models, and suitable for radiotracers with slow kinetics [17].

**Fig. 2:**
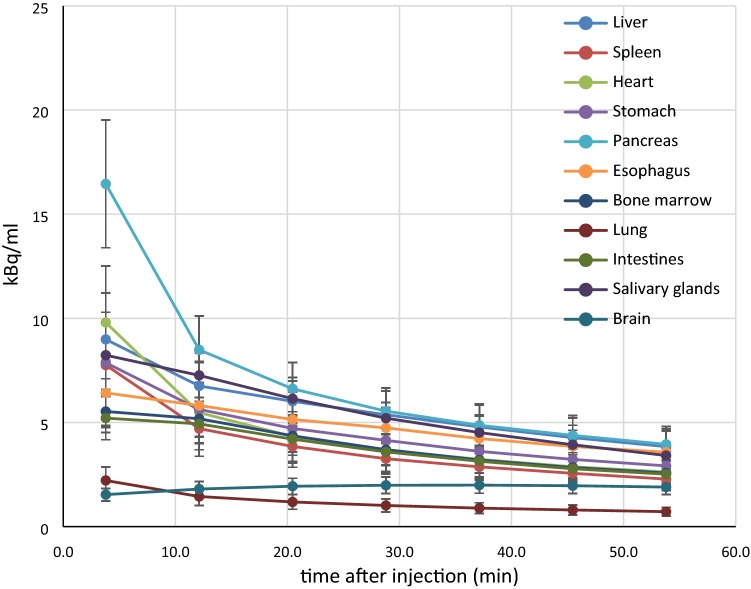
Averaged ^18^F-FBPA time-activity curve in kBq/ml, in all selected VOIs. A gradual washout is observed in all organs from the beginning of the study, except for the brain. The highest uptake is seen in the pancreas, and the lowest is seen in the lung. Each time point matches the middle of each scan. In the brain continuous increase of radioactivity up to thirty-forth minute and slow decrease until the end of the study is observed

Brain time-activity curve had significantly different pattern compared to other organs (Fig. 2), with the gradual increase of radioactivity up to thirty-fourth minute and following slow decrease until the end of the study. Probably it is caused by different pharmacokinetics of the tracer due to high Large Amino Acid Transporter type 1 (LAT1) expression in the blood–brain barrier [19]. Thereby Ichise MA2 could be the optimal model for this area.

Maximal Vt value did not exceed 1.08 ml/ml (in the pancreas). This value is much lower than Vt values for tumors, that could be found in literature for animal tumor model [20], or derived from published rate constants for human and rat glioma [21, 22], reflecting lower *k*_1_/*k*_2_ ratio in normal tissues, that in turn could be explained by differences in expression of amino acid transporters, between normal and tumor cells and their affinity to the ligand. In tumors LAT1 and ATB^0,+^ upregulation has been found, and these transporters are responsible for ^10^B-BPA and ^18^F-FBPA uptake in tumor cells [23]. LAT2, which is expressed in normal cells, has lower affinity for ^18^F-FBPA, comparing to LAT1 and shows lower ^18^F-FBPA transportability [24].

In addition, mean Vt appeared to be lower than 1 for all selected organs, suggesting tracer concentration in normal tissues to be lower than that in plasma, after the equilibration time.

There are several factors that could influence the accuracy of acquired Vt values. Areas selected for analysis included functionally and histologically different tissues. These tissues contain boron compound not only in the interstitium but also in blood vessels, as well as chyme and gases in the hollow organs and air in the lungs, which affects the accuracy of boron concentration measurements. The influence of such factors was not evaluated in the current study.

Due to the superimposition of the pancreas VOI and area of high tracer accumulation in the left kidney, the tail of the pancreas was partially excluded from the analysis.

Also, ^18^F-FBPA metabolization with the detachment of the boron atom takes place to some degree, most likely due to hepatic enzymes' activity [25], thereby contributing to the bias of measurements.

System L is the transporting system responsible for ^10^B-BPA uptake in normal tissues [26], and LAT2 is l-amino acid transporter mainly expressed in normal cells [23, 27]. However, ^18^F-FBPA had lower affinity and transportability to LAT2 in comparison with ^10^B-BPA [24], which could also have an impact on the results.

It was found that N/B ratios for ^10^B-BPA and ^19^F labeled FBPA differed depending on the administration protocol and were lower for the continuous infusion group comparing to the single-injection group [11]. Although the result is obtained in the animal experiment and compounds were administrated subcutaneously, whereas intravenous infusion is commonly used in humans, it still can cause the discrepancy between ^10^B concentration values depending on the protocol features.

In another study of ^10^B-BPA pharmacokinetics, biphasic exponential clearance of ^10^B-BPA concentration from the blood after the peak is reached was reported, and the time lag between the ^10^B-BPA blood and tissue concentrations was suggested [28]. The equilibrium state between these concentrations will be achieved during the late phase of the clearance. However, if neutron irradiation takes place before the late phase equilibrium reached, disagreement between actual and estimated values could occur.

In summary, the current research attempted to evaluate ^10^B concentrations in vivo using pharmacokinetic analysis. The ^10^B concentration in normal tissues was best estimated using Vt values of ^18^F-FBPA with the Ichise multilinear analysis MA2.

## Electronic supplementary material

Below is the link to the electronic supplementary material.

Supplementary material 1 (PDF 36 kb)

## References

[1] Lin YC, Hwang JJ, Wang SJ, Yang BH, Chang CW, Hsiao MC (2012). Macro- and microdistributions of boron drug for boron neutron capture therapy in an animal model. Anticancer Res.

[2] Haritz D, Gabel D, Huiskamp R (1994). Clinical Phase-I study of Na2B12H11SH (BSH) in patients with malignant glioma as precondition for boron neutron capture therapy (BNCT). Int J Radiat Oncol Biol Phys.

[3] Mallesch JL, Moore DE, Allen BJ, McCarthy WH, Jones R, Stening WA (1994). The pharmacokinetics of *p*-boronophenylalanine fructose in human patients with glioma and metastatic melanoma. Int J Radiat Oncol Biol Phys.

[4] Coderre JA, Chanana AD, Joel DD, Elowitz EH, Micca PL, Nawrocky MM (1998). Biodistribution of boronophenylalanine in patients with glioblastoma multiforme: boron concentration correlates with tumor cellularity. Radiat Res.

[5] Kubota R, Yamada S, Ishiwata K, Tada M, Ido T, Kubota K (1993). Cellular accumulation of ^18^F-labelled boronophenylalanine depending on DNA synthesis and melanin incorporation: a double-tracer microautoradiographic study of B16 melanomas in vivo. Br J Cancer.

[6] Yoshida F, Matsumura A, Shibata Y, Yamamoto T, Nakauchi H, Okumura M (2002). Cell cycle dependence of boron uptake from two boron compounds used for clinical neutron capture therapy. Cancer Lett.

[7] Fairchild RG, Bond VP (1985). Current status of ^10^B-neutron capture therapy: enhancement of tumor dose via beam filtration and dose rate, and the effects of these parameters on minimum boron content: a theoretical evaluation. Int J Radiat Oncol Biol Phys.

[8] Barth RF, Coderre JA, Vicente MG, Blue TE (2005). Boron neutron capture therapy of cancer: current status and future prospects. Clin Cancer Res.

[9] Kabalka GW, Smith GT, Dyke JP, Reid WS, Longford CP, Roberts TG (1997). Evaluation of fluorine-18-BPA-fructose for boron neutron capture treatment planning. J Nucl Med.

[10] Hanaoka K, Watabe T, Naka S, Kanai Y, Ikeda H, Horitsugi G (2014). FBPA PET in boron neutron capture therapy for cancer: prediction of ^10^B concentration in the tumor and normal tissue in a rat xenograft model. EJNMMI Res.

[11] Watanabe T, Hattori Y, Ohta Y, Ishimura M, Nakagawa Y, Sanada Y (2016). Comparison of the pharmacokinetics between L-BPA and L-FBPA using the same administration dose and protocol: a validation study for the theranostic approach using [^18^F]-L-FBPA positron emission tomography in boron neutron capture therapy. BMC Cancer.

[12] Shimosegawa E, Isohashi K, Naka S, Horitsugi G, Hatazawa J (2016). Assessment of ^10^B concentration in boron neutron capture therapy: potential of image-guided therapy using ^18^FBPA PET. Ann Nucl Med.

[13] Watabe T, Hanaoka K, Naka S, Kanai Y, Ikeda H, Aoki M (2017). Practical calculation method to estimate the absolute boron concentration in tissues using ^18^F-FBPA PET. Ann Nucl Med.

[14] Isohashi K, Shimosegawa E, Naka S, Kanai Y, Horitsugi G, Mochida I (2016). Comparison of the image-derived radioactivity and blood-sample radioactivity for estimating the clinical indicators of the efficacy of boron neutron capture therapy (BNCT): 4-borono-2-^18^F-fluoro-phenylalanine (FBPA) PET study. EJNMMI Res.

[15] Ishiwata K, Ido T, Mejia AA, Ichihashi M, Mishima Y (1991). Synthesis and radiation dosimetry of 4-borono-2-[^18^F]fluoro-d,l-phenylalanine: a target compound for PET and boron neutron capture therapy. Int J Rad Appl Instrum A.

[16] Logan J, Fowler JS, Volkow ND, Wolf AP, Dewey SL, Schlyer DJ (1990). Graphical analysis of reversible radioligand binding from time-activity measurements applied to [N-^11^C-methyl]-(–)-cocaine PET studies in human subjects. J Cereb Blood Flow Metab.

[17] Ichise M, Toyama H, Innis RB, Carson RE (2002). Strategies to improve neuroreceptor parameter estimation by linear regression analysis. J Cereb Blood Flow Metab.

[18] Watabe H, Ikoma Y, Kimura Y, Naganawa M, Shidahara M (2006). PET kinetic analysis—compartmental model. Ann Nucl Med.

[19] Boado RJ, Li JY, Nagaya M, Zhang C, Pardridge WM (1999). Selective expression of the large neutral amino acid transporter at the blood–brain barrier. Proc Natl Acad Sci USA.

[20] Grunewald C, Sauberer M, Filip T, Wanek T, Stanek J, Mairinger S (2017). On the applicability of [^18^F]FBPA to predict L-BPA concentration after amino acid preloading in HuH-7 liver tumor model and the implication for liver boron neutron capture therapy. Nucl Med Biol.

[21] Imahori Y, Ueda S, Ohmori Y, Kusuki T, Ono K, Fujii R (1998). Fluorine-18-labeled fluoroboronophenylalanine PET in patients with glioma. J Nucl Med.

[22] Chen JC, Chang SM, Hsu FY, Wang HE, Liu RS (2004). MicroPET-based pharmacokinetic analysis of the radiolabeled boron compound [^18^F]FBPA-F in rats with F98 glioma. Appl Radiat Isot.

[23] Wongthai P, Hagiwara K, Miyoshi Y, Wiriyasermkul P, Wei L, Ohgaki R (2015). Boronophenylalanine, a boron delivery agent for boron neutron capture therapy, is transported by ATB^0,+^, LAT1 and LAT2. Cancer Sci.

[24] Watabe T, Ikeda H, Nagamori S, Wiriyasermkul P, Tanaka Y, Naka S (2017). (18)F-FBPA as a tumor specific tracer of L-type amino acid transporter 1 (LAT1): PET evaluation in tumor and inflammation compared to (18)F-FDG and (11)C-methionine. Eur J Nucl Med Mol Imaging.

[25] Ishiwata K, Ido T, Kawamura M, Kubota K, Ichihashi M, Mishima Y (1991). 4-Borono-2-[^18^F] fluoro-d,l-phenylalanine as a target compound for boron neutron capture therapy: tumor imaging potential with positron emission tomography. Int J Rad Appl Instrum B.

[26] Wittig A, Sauerwein WA, Coderre JA (2000). Mechanisms of transport of *p*-borono-phenylalanine through the cell membrane in vitro. Radiat Res.

[27] Nakada N, Mikami T, Hana K, Ichinoe M, Yanagisawa N, Yoshida T (2014). Unique and selective expression of L-amino acid transporter 1 in human tissue as well as being an aspect of oncofetal protein. Histol Histopathol.

[28] Kiger WS, Palmer MR, Riley KJ, Zamenhof RG, Busse PM (2001). A pharmacokinetic model for the concentration of ^10^B in blood after boronophenylalanine-fructose administration in humans. Radiat Res.

