# Supplementary material for: Evaluation of the total distribution volume of 18F-FBPA in normal tissues of healthy volunteers by non-compartmental kinetic modeling
Source: Ann Nucl Med. 2019 Dec 5;34(3):155–62. doi: 10.1007/s12149-019-01427-9 (PMC7033086; doi:10.1007/s12149-019-01427-9)
Supplement: Supplementary file 1 — Supplementary material 1 (PDF 36 kb) [file 12149_2019_1427_MOESM1_ESM.pdf]

Article title: “Evaluation of the total distribution volume of ^{18}F-FBPA in normal tissues of healthy volunteers by non-compartmental kinetic modeling”**.**

Authors: Victor Romanov., Kayako Isohashi., Galal Al-Obthani., Rouaa Beshr., Genki Horitsugi., Yasukazu Kanai., Sadahiro Naka., Tadashi Watabe., Eku Shimosegawa., Jun Hatazawa.

Affiliation and address of the corresponding author: Jun Hatazawa; Yamada Oka 2-2, Suita City, Osaka 565-0871, Japan; e-mail: hatazawa@tracer.med.osaka-u.ac.jp

Journal name: Annals of Nuclear Medicine (ANM).

Online Resource1 Interpolated plasma activity curves for all subjects in kBq/ml, derived from the clearance data points of actual plasma activity with a bi-exponential model. Interpolated curve is colored in red, and actual plasma curve is colored in blue.

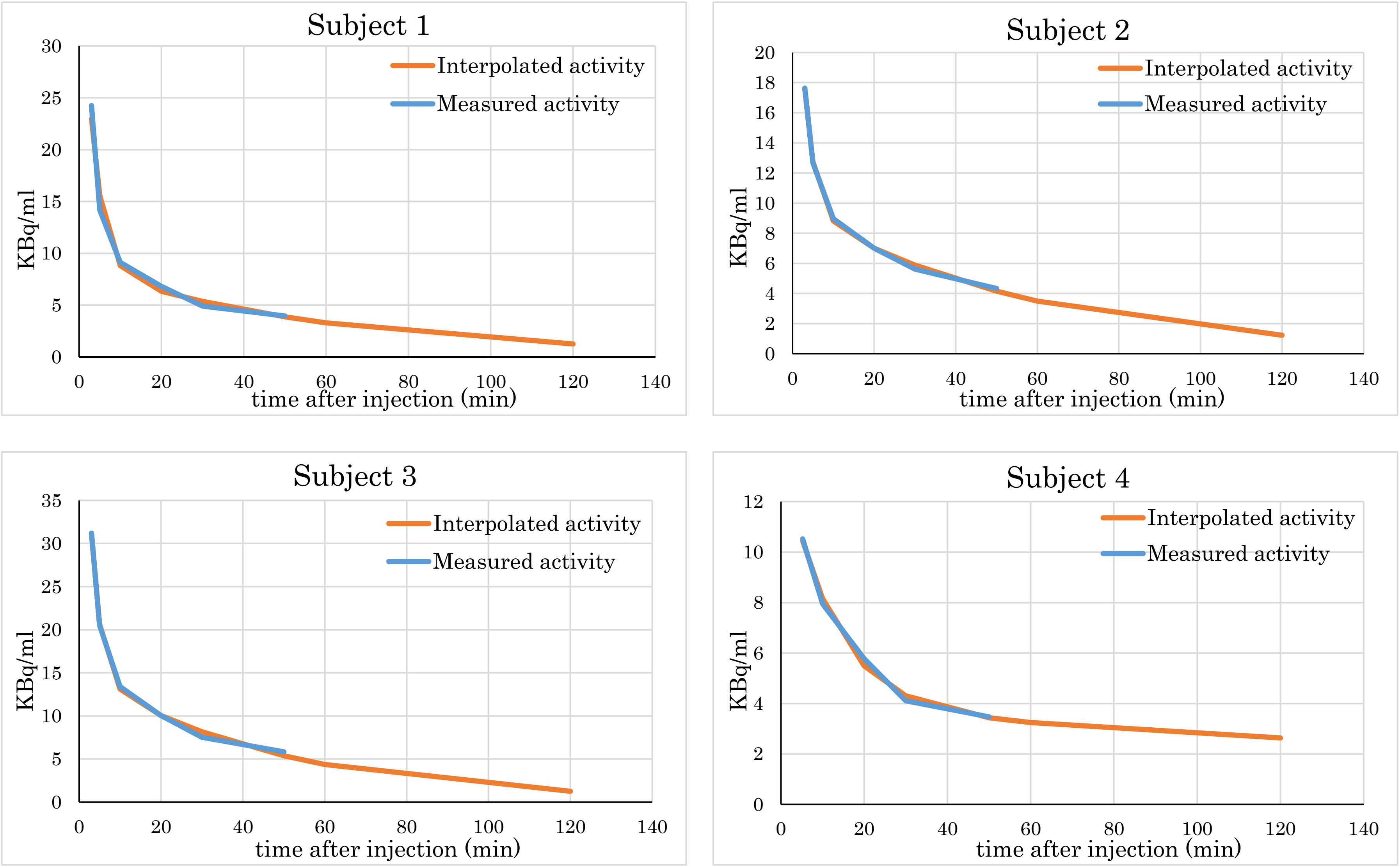
Subject 1
Interpolated activity
Measured activity
KBq/ml
0
5
10
15
20
25
30
0
20
40
60
80
100
120
140
time after injection (min)
Subject 2
Interpolated activity
Measured activity
KBq/ml
0
2
4
6
8
10
12
14
16
18
20
0
20
40
60
80
100
120
140
time after injection (min)
Subject 3
Interpolated activity
Measured activity
KBq/ml
0
5
10
15
20
25
30
35
0
20
40
60
80
100
120
140
time after injection (min)
Subject 4
Interpolated activity
Measured activity
KBq/ml
0
2
4
6
8
10
12
0
20
40
60
80
100
120
140
time after injection (min)

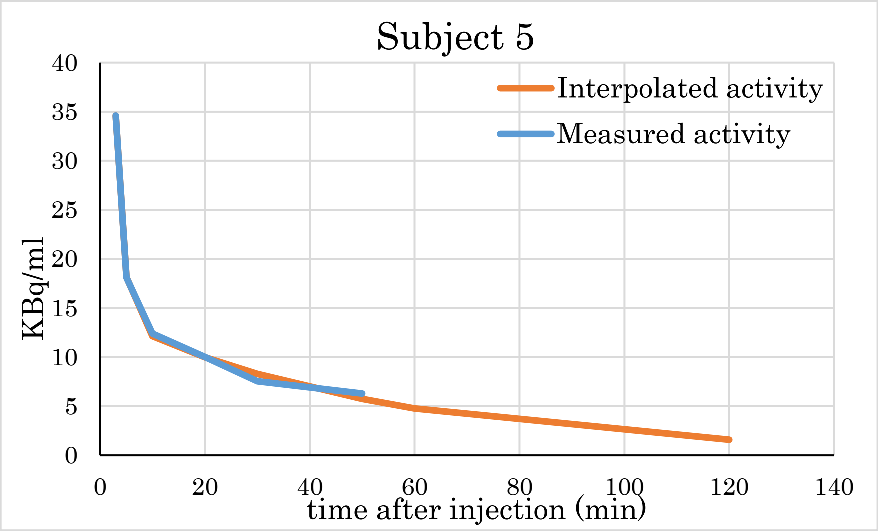
Subject 5
Interpolated activity
Measured activity
KBq/ml
0
5
10
15
20
25
30
35
40
0
20
40
60
80
100
120
140
time after injection (min)

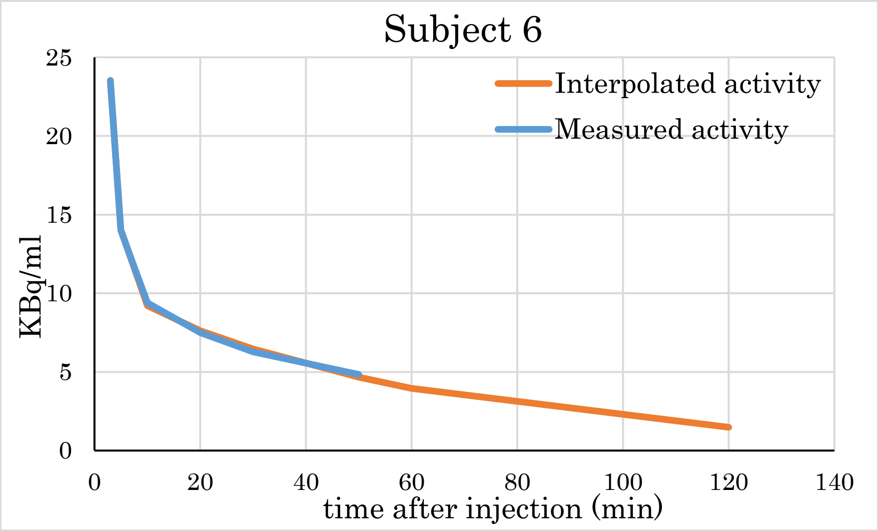
Subject 6
Interpolated activity
Measured activity
KBq/ml
0
5
10
15
20
25
0
20
40
60
80
100
120
140
time after injection (min)
